# LRET-based intramolecular distance measurements in LeuT_Aa_

**DOI:** 10.1186/1471-2210-11-S2-A21

**Published:** 2011-09-05

**Authors:** Azmat Sohail, Peggy Stolt-Bergner, Gerhard F Ecker, Michael Freissmuth, Thomas Stockner, Harald H Sitte, Walter Sandtner

**Affiliations:** 1 Institute of Pharmacology, Center of Physiology and Pharmacology, Medical University of Vienna, 1090 Vienna, Austria; 2 Research Institute of Molecular Pathology, Campus Vienna Biocenter, 1030 Vienna, Austria; 3 Department of Medicinal Chemistry, University of Vienna, 1090 Vienna, Austria

## Background

LeuT_Aa_ is a bacterial orthologue of mammalian Solute Carrier Class 6 (SLC6) neurotransmitter transporters from *Aquifex aeolicus* which transports leucine and alanine. SLC6 transporters are of great pharmacological interests because of their crucial role in neurotransmitter clearance. These proteins are also targets of many clinically relevant drugs. The crystal structure of LeuT_Aa_ has been resolved at atomic resolution of ~1.5 Å. Although LeuT has a low overall sequence identity of about 20–25% to SLC6 members, the grade of conservation reaches around 55% in functionally critical transmembrane domains 1, 3, 6 and 8. For this very reason we are using LeuT_Aa_ as a good structural paradigm to explore the structural/functional information about SLC6-family members.

## Methods and results

In order to explore structural/functional information about the SLC6 family, we initiated a study to measure intramolecular distance changes associated with substrate transport by Luminescence Resonance Energy Transfer (LRET). LRET is based on the Förster effect and relies on the non-radiative transfer of energy from a donor element to an acceptor fluorophore. We introduce Lanthanide-binding Tags (LBT) at selected positions into the LeuT to accommodate the excitable donor terbium along with cysteines, where acceptor fluorophores are attached. After expression and purification of these mutants, we measure the distances between donor and acceptor at atomic resolution. In order to screen the mutants of LeuT_Aa_ for their function, we have recently established the scintillation proximity assay (SPA). To date, we screened a number of LBT-mutants as well as cysteine-mutants for their function; several have been shown to be functional and will be tested now in the LRET setup.

## Conclusion and future plan

Since we obtained functional LBT- and cysteine-mutants we are looking forward to measure distances between several terbium-bound LBTs and cysteine-bound fluorophores. We will force the transporter into an outward- or inward-facing state and validate a 3D-model for LeuT.

